# The Role of Interleukin-17 in Lung Cancer

**DOI:** 10.1155/2016/8494079

**Published:** 2016-10-30

**Authors:** Feng Wu, Juanjuan Xu, Qi Huang, Jieli Han, Limin Duan, Jinshuo Fan, Zhilei Lv, Mengfei Guo, Guorong Hu, Lian Chen, Shuai Zhang, Xiaonan Tao, Wanli Ma, Yang Jin

**Affiliations:** Key Laboratory of Respiratory Diseases, Ministry of Health, Department of Respiratory and Critical Care Medicine, Union Hospital, Tongji Medical College, Huazhong University of Science and Technology, 1277 Jiefang Avenue, Wuhan 430022, China

## Abstract

Tumour-associated inflammation is a hallmark of malignant carcinomas, and lung cancer is a typical inflammation-associated carcinoma. Interleukin-17 (IL-17) is an important inflammatory cytokine that plays an important role in chronic inflammatory and autoimmune diseases and in inflammation-associated tumours. Numerous studies have shown that IL-17 directly or indirectly promotes tumour angiogenesis and cell proliferation and that it inhibits apoptosis via the activation of inflammatory signalling pathways. Therefore, IL-17 contributes to the metastasis and progression of lung cancer. Research advances with respect to the role of IL-17 in lung cancer will be presented as a review in this paper.

## 1. Introduction

Due to its high morbidity and mortality rates, lung cancer is currently one of the most common malignant carcinomas and has become a “deadly disease” among malignant carcinomas in China and the rest of the world [1, 2]. Metastasis, which is one of the most significant features of tumours, is the major cause of lung cancer relapse, poor prognosis, and death in patients with this disease [3, 4].

Researchers have been studying the relationship between chronic inflammation and cancer for many years [5]. As early as 1863, Rudolph Virchow, a pathologist, discovered that tumour development was closely related to inflammation, and numerous subsequent studies confirmed his conclusion [6]. In 2011, Hanahan and Weinberg proposed that the inflammatory microenvironment of tumours (i.e., the local environment formed by tumour cells, endothelial cells, fibroblasts, infiltrating inflammatory cells, and extracellular matrix) should be considered as “the seventh feature of tumours” [7]. According to the following four aspects, lung cancer has also been demonstrated to be a typical inflammation-associated carcinoma: (1) lung cancer is the deadliest carcinoma in the world; epidemiological surveys have shown that lung cancer is related to smoking, exposure to certain carcinogens, and individual genetic predisposition [8]. In addition, nearly 100% of lung cancer cases in nonsmokers are closely related to lung inflammation [9]. (2) Many chronic inflammatory pulmonary diseases have a pathogenesis similar to that of lung cancer and some of these diseases such as chronic obstructive pulmonary disease, chronic infections (e.g.,* Mycobacterium tuberculosis* and* Chlamydia* infections), and occupational lung disease (e.g., asbestos- and silica-induced pulmonary fibrosis and inflammation) may progress to lung cancer under certain conditions. These lung diseases have demonstrated a close relationship with lung cancer [10]. (3) Studies have shown that nonsteroidal anti-inflammatory drugs (e.g., aspirin) and other anti-inflammatory drugs can reduce the incidence of lung cancer [11]. (4) The lung cancer inflammation index may be used to assess the prognosis of individuals with metastatic nonsmall cell lung cancer (NSCLC) [12]. Tumour cells in the lung cancer microenvironment, as well as cytokines, chemokines, and inflammatory mediators secreted by interstitial cells, interact with and act upon target cells to activate progrowth and proinflammatory signalling pathways; this interaction promotes the malignant biological behaviour of lung cancer and tumour progression. Numerous studies have shown that the proinflammatory cytokine IL-17 directly or indirectly promotes tumour angiogenesis and cell proliferation and that it inhibits apoptosis via the activation of inflammatory signalling pathways; IL-17 therefore contributes to the progression of lung cancer. The present review will illustrate advances in research in terms of the role of IL-17 in lung cancer.

## 2. Overview of IL-17 

IL-17 was first discovered by Rouvier et al. [13] while they screened a cDNA library of mouse lymphoid cells. This cytokine was initially named cytotoxic T lymphocyte antigen 8 (CTLA-8). Yao et al. [14] confirmed that CTLA-8 is a cytokine derived from CD4+ T cells and thus named it IL-17. Since then, other cytokines that belong to this new family of cytokines (including 6 cytokines: IL-17 (as the founding cytokine), IL-17B, IL-17C, IL-17D, IL-17E (also known as IL-25), and IL-17F, as well as five receptors: IL-17RA, IL-17RB, IL-17RC, IL-17RD, and IL-17RE) have been discovered [15]; of these, the heterodimer of IL-17RA and IL-17RC serves as a coreceptor of IL-17A and IL-17F, and the roles of both IL-17RA and IL-17RC are essential in disease processes [16, 17]. IL-17 and T helper 17 (Th17) cells have differing roles in tumour progression, and researchers have reached different conclusions about the role of IL-17 in tumours [18–23]. This is because IL-17 is derived from multiple sources in the body, binds to a number of complex receptors, activates a wide range of signalling pathways, and has diverse target cells. In regard to lung cancer, in addition to Th17 cells, a multitude of immune cells such as neutrophils [24], mononuclear cells, and *γδ*T cells [19, 25] infiltrate the tumour; even lung cancer cells [26] may secrete IL-17. In the sections below, we will focus on the role of IL-17 in lung cancer in detail.

## 3. The Effect of IL-17 on the Tumorigenesis of Lung Cancer

Studies have shown that smoking is related to polymorphisms in the IL-17 gene but that smoking is unrelated to those in IL-17F. Smokers who carry at least one copy of the IL-17 G-152A allele have a 2.06-fold higher risk of lung cancer [27]. One IL-17 gene polymorphism also upregulates IL-17 expression, which enhances susceptibility to lung cancer [28, 29]. Animal models have shown that the incidence rate of lung cancer was significantly lower in IL-17 knockout mice with a lung-specific K-ras mutation than in mice with a local pulmonary K-ras mutation [30]. In addition, Xu et al. [23] used an adenovirus that overexpressed IL-17 cDNA to enhance pulmonary expression of IL-17 in mice with K-ras mutations. The results showed that 1 week after adenovirus inhalation, pulmonary IL-17 expression (150-fold difference) and the incidence of lung cancer were significantly higher in the experimental group than in the control group; moreover, high IL-17 expression was associated with upregulated expression of matrix metallopeptidase 9 (MMP-9) and higher invasiveness of tumour cells. These results directly and indirectly confirm that IL-17 promotes the development of lung cancer.

## 4. The Effect of IL-17 on the Proliferation and Apoptosis of Lung Cancer Cells

Numasaki et al. [24] showed that neither exogenous IL-17 stimulation nor IL-17cDNA transfection affected in vitro cell proliferation of the nonsmall cell lung cancer cell lines Sq-19 and A549. However, in vivo animal models showed that mice with severe combined immune deficiency (SCID) that were inoculated with IL-17cDNA-transfected Sq-19 and A549 cells showed a higher tumour volume than the vehicle control group. In contrast, the Ki-67 index remained unchanged, and results of a terminal deoxynucleotidyl transferase dUTP nick end labelling (TUNEL) assay suggested a lower level of apoptosis. This indicates that IL-17 affects lung cancer progression via the inhibition of NSCLC cell apoptosis rather than by the promotion of cell proliferation. Li et al. [31] performed cell counts and found that IL-17A did not promote 95C or 95D cell proliferation in vitro. However, Ye et al. [32] performed flow cytometry and found that IL-17 inhibited apoptosis through the promotion of the proliferation of A549 and SK-MES-1 cells (Ki-67 detection) via the signal transducer and activator of transcription 3 (STAT3) pathway. The differences in these in vitro studies may be related to the study methods and laboratory conditions.

## 5. IL-17 Promotes Angiogenesis and Lymphangiogenesis of Lung Cancer

Immunohistochemical staining of serial sections of clinical human lung cancer specimens for IL-17 and CD31 or CD34 showed that high IL-17 expression is positively correlated with tumour microvessel density (MVD) [24, 33]. Other studies showed that high IL-17 expression is positively correlated with vascular endothelial growth factor-C (VEGF-C) and VEGF-D expression and with high microlymphatic vessel density (LVD) [34]. Many studies have suggested that the mechanism by which IL-17 promotes angiogenesis or lymphangiogenesis is related to VEGF and its receptor family. Brussino et al. [35] showed that IL-17 expression in exhaled breath condensate from patients with NSCLC was correlated with VEGF-A expression. Lin et al. [36] confirmed that in lung cancer patients, serum IL-17 was positively correlated with VEGF-A expression. Li et al. [31] conducted an in vitro study and found that IL-17A affected VEGF-A expression in 95D and 95C cells in a dose-dependent manner; moreover, supernatant from IL-17A-pretreated 95D and 95C cells significantly promoted endothelial angiogenesis. Li et al. [37] confirmed that IL-17 promoted VEGF-A expression in A549 cells. However, Numasaki et al. [24] revealed that IL-17 promoted the secretion of CXC chemokine receptor 1 (CXCL1), CXCL5, CCR6, and CXCL8 in Sq-19 and A549 cells but that IL-17 had no effect on VEGF expression; furthermore, conditioned medium from IL-17-stimulated Sq-19 and A549 cells promoted chemotactic activity of endothelial cells. Numasaki et al. [24] used a mouse model and found stronger CD31 staining in severe combined immunodeficient (SCID) mice that were inoculated with IL-17 cDNA-transfected Sq-19 and A549 cells. The expression of angiogenic factors such as CXCL1, CXCL5, and CXCL8 was increased in the peripheral blood mice that overexpressed IL-17, whereas CXCR-2 blocked the angiogenic effects of IL-17, which suggests that IL-17 promoted tumour angiogenesis via CXCR-2. Therefore, studies have differed in their conclusions about the effect of IL-17 on VEGF-A expression in A549 cells. In addition, Chen et al. [38] confirmed that IL-17 contributed to the metastasis of NSCLC via the promotion of lymphangiogenesis; this was achieved through the promotion of VEGF-C (an important cytokine for lymphangiogenesis) secretion by the Lewis lung carcinoma (LLC) cell line (mouse) and A549 cells (human) via the extracellular signal-regulated protein kinase 1/2 (ERK 1/2) pathway and through chemotactic effects on lymphatic epithelial cells.

## 6. IL-17 Promotes the Metastasis of Lung Cancer

Studies have shown that IL-17 promotes the metastasis of lung cancer through several pathways. Studies of clinical human NSCLC specimens have shown that IL-17 [34] and IL-17RA [37] are correlated with the metastatic status of lymph nodes. Lung cancer patients with high serum IL-17 expression have a significantly higher risk of brain metastasis, and the IL-17 level in cerebrospinal fluid was significantly higher in lung cancer patients with brain metastasis than in those without brain metastasis; this suggests that IL-17 may play an important role in the metastasis of lung cancer to the brain [39]. Numasaki et al. [24] showed that IL-17 was unrelated to lymph node metastasis, and this difference may be due to sample size and the specificity of the specimens. Studies have shown that lung metastases occurred significantly less frequently in an IL-17 knockout mouse model of lung adenocarcinoma than in wild-type mice after intravenous injection of the mouse LLC cell line into the tail vein [37, 40]. Li et al. [37] investigated the mechanism of IL-17 in the promotion of lung cancer metastasis. They found STAT3 phosphorylation and a decrease in MMP9 expression in an IL-17 knockout mouse model of lung adenocarcinomas that involved the inoculation of mice with LLC cells via the tail vein. Carmi et al. [40] established a mouse model of lung cancer metastasis by injection of LLC cells into the tail vein and confirmed that, in the tumour microenvironment, IL-1 recruited *γδ*T cells to secrete IL-17, which promoted metastasis. In addition, IL-17 expression was negatively correlated with the Th1 cell transcription factor T-bet, whereas T-bet inhibited IL-17 expression and tumour progression. The use of an anti-IL-17 antibody also delayed the progression of lung metastasis in a T-bet knockout mouse model of lung adenocarcinoma after the injection of LLC cells into the tail vein [41].

Ye et al. [32] conducted an in vitro study with NSCLC cell lines and showed that IL-17 promoted migratory activity of A549 and SK-MES-1 cells via the STAT3 pathway. Our study and the study by Li et al. [37] showed that IL-17 promoted the migration and metastasis of A549 and LLC cells via the STAT3 pathway. Other studies have also demonstrated that IL-17 promoted the migration of A549 cells via the NF-*κ*B pathway [42]. Furthermore, epithelial-mesenchymal transition (EMT) is another important mechanism in the process of tumour metastasis [43], including that of lung cancer [44–48]. One study reported that IL-17 induced changes in the expression of EMT-related markers in the lung cancer-derived cell line A549. For instance, IL-17 induced high expression of the mesenchymal cell surface marker vimentin while it inhibited the expression of the epithelial cell surface marker E-cadherin, which enhanced the invasiveness of A549 cells. Further analysis showed that IL-17 activated the NF-*κ*B pathway and induced the expression of the EMT-associated transcription factor ZEB1, which contributed to the occurrence of EMT; this suggests that IL-17 promoted the metastasis of lung cancer-derived A549 cells [42]. In addition, IL-17 promoted MMP2 and MMP9 secretion by A549 cells [23, 37, 49], which promoted the invasion and metastasis of lung cancer. These results suggest that IL-17 promotes the metastasis of lung cancer via several different pathways.

## 7. The Effect of IL-17 on Immune Tolerance of Lung Cancer

The immune microenvironment of tumours plays an important role in tumour progression. In lung cancer, IL-17 is derived from different sources within the microenvironment. This allows tumour cells to develop immune tolerance to different types of cells of the immune system, which may either promote or inhibit the progression of lung cancer. IL-17 mediates and supports the immune microenvironment of tumours. Myeloid-derived suppressor cells (MDSCs) are precursors of granulocytes, macrophages, dendritic cells, and other immune cells. IL-17 expression in the tumour microenvironment helps to recruit MDSCs, which leads to the inhibition of the body's antitumour immune response [30, 50]. Moreover, IL-17 recruits M2 tumour-associated macrophages (TAMs) into the microenvironment of lung tumours [51] and not only does high IL-17 expression in the microenvironment of lung tumours recruit TAMs, but it also upregulates cyclooxygenase 2 (COX2) expression in lung cancer cells. This increases prostaglandin E2 (PGE2) synthesis, which in turn promotes the differentiation of M2 TAMs [52], forming a vicious cycle. Furthermore, IL-17 mediates and promotes the immune microenvironment of tumour suppression. Ye et al. [53] confirmed that in human malignant pleural effusion, CCL20 and CCL22 recruited Th17 cells, and then IL-1*β*, IL-6, and IL-23 induced the differentiation and maturation of Th17 cells, thus inhibiting tumour progression. Lin et al. [54] used an LLC mouse model of malignant pleural effusion and showed that tumours progressed faster and that survival was significantly shorter in IL-17 knockout mice. In addition, the lack of IL-17 inhibited the activation of the STAT1 pathway and promoted the activation of Th1 cells, whereas the lack of IFN*γ* inhibited the activation of the STAT3 pathway, promoted activation of Th17 cells, and inhibited the formation of malignant pleural effusion; these events prolonged the survival of these mice. Marshall et al. [55] showed that IL-17 was required for the efficacy of combination treatment of LLC in mice that were given a PI3K pathway inhibitor and a toll-like receptor agonist. In addition, in lung cancer xenografts, IL-17 derived mainly from *γδ*T cells [40] played an antitumour role. Cheng et al. showed that the stimulation of symbiotic bacteria within organisms helped to maintain *γδ*T cell activation, which maintained the antitumour effects of these cells [56]. Thus, IL-17 is directly or indirectly involved in the immunomodulation of lung cancer.

## 8. The Role of IL-17 in the Clinical Staging, Diagnosis, and Prognosis of Lung Cancer

The early diagnosis of lung cancer significantly reduces morbidity and mortality rates. IL-17 exerts complex effects on tumour progression, and a growing body of evidence suggests that IL-17 is an important marker for the diagnosis and prognosis of lung cancer and provides an important basis for the differentiation of benign tumours from malignant tumours. Zhang et al. [21] and Zeng et al. [22] conducted meta-analyses and showed that high IL-17 expression was significantly correlated with an advanced stage of NSCLC (III/IV), overall survival (OS), and disease-free survival (DFS), which suggests that IL-17 promotes the growth and development of NSCLC. Chen et al. [34] performed immunohistochemical staining to detect IL-17 in human NSCLC tissues and found that high IL-17 expression was significantly correlated with the clinical and pathological features of patients (e.g., smoking status and TNM staging), LVD, OS, and DFS. Moreover, univariate and multivariate analyses suggested that IL-17 was an independent predictor of the OS and DFS of patients with NSCLC. Zhang et al. [57] showed that high IL-17 expression was significantly correlated with the median survival of patients with NSCLC. To initiate proper treatment of patients with pleural effusion, it is critical to determine whether the pleural effusion is benign [58–60]. Malignant pleural effusion is primarily diagnosed by pathological confirmation of tumour cells in the pleural fluid; however, the chance of detecting tumour cells by pleural effusion cytology (the preferred diagnostic method) and biopsy is low and the sensitivity is only 30% to 60% [61]. In numerous recent studies, an enzyme-linked immunosorbent assay (ELISA) was performed, which showed that the IL-17 level was significantly higher in pleural effusion compared with peripheral blood in patients with lung cancer [60, 62, 63]. As an alternative to the cytological assay, the diagnostic sensitivity of ELISA for malignant pleural effusion is as high as 76.8%, which may increase to 96.4% when combined with other markers, such as carcinoembryonic antigen (CEA) [37, 58, 60]. An immunoassay of surgical lung cancer specimens showed that IL-17 was significantly higher in tumour tissues than in adjacent tissues and distant normal lung tissue [34]. Moreover, many studies have shown that the IL-17 level in peripheral blood was significantly higher in lung cancer patients than in healthy individuals [31, 37, 64].

## 9. IL-17 as a Treatment Target

IL-17 is not only a potential diagnostic marker for lung cancer but also a new treatment target. Chung et al. [50] observed subcutaneous xenografts of lung cancer, colon cancer, and lymphoma as well as orthotopic xenografts of lung cancer. They found that Th17 cells in tumour tissue secreted IL-17, upregulated the expression of granulocyte colony stimulating factor via the NF-*κ*B pathway, recruited MDSCs to the tumour microenvironment, and led to resistance to anti-VEGF treatment. In contrast, anti-VEGF treatment in an IL-17 receptor knockout mouse xenograft model and anti-VEGF combined with anti-IL-17 treatment in normal mice inhibited tumour resistance to antiangiogenic treatment. As we deepen our understanding of the function of IL-17 in lung cancer, anti-IL-17 treatment will play an important role in the treatment of lung cancer and may become a new option for the treatment of patients with lung cancer [65].
